# Potential benefits of vitamin D for sepsis prophylaxis in critical ill patients

**DOI:** 10.3389/fnut.2023.1073894

**Published:** 2023-04-04

**Authors:** Jianbin Guan, Maoyou Shichen, Zhihui Liang, Shuang Yu, Min Zhao, Lu Zhang, Ronggui Lv, Yong Liu, Ping Chang, Zhanguo Liu

**Affiliations:** ^1^Department of Critical Care Medicine, Zhujiang Hospital, Southern Medical University, Guangzhou, Guangdong, China; ^2^Department of Intensive Care Unit, Guangdong Provincial People’s Hospital Nanhai Hospital, Foshan, Guangdong, China; ^3^Guangzhou First People’s Hospital, Guangzhou, Guangdong, China; ^4^Department of Intensive Care Unit, Shenzhen Hospital, Southern Medical University, Shenzhen, China

**Keywords:** vitamin D, sepsis prophylaxis, suspected infection, critical ill patients, critical care

## Abstract

**Background:**

Vitamin D deficiency is common in critically ill patients with suspected infection and is strongly associated with the predisposition of sepsis and a poor prognosis. The effectiveness of vitamin D supplementation for preventing sepsis remains unclear. This retrospective cohort study investigated the effect of vitamin D supplementation on sepsis prophylaxis in critically ill patients with suspected infection.

**Methods:**

This retrospective cohort study included 19,816 adult patients with suspected infection in intensive care units (ICU) from 2008 to 2019 at the Beth Israel Deaconess Medical Center, Boston, USA. The included patients were divided into the vitamin D cohort or non-vitamin D cohort according to vitamin D administration status. The primary outcomes were the incidence of sepsis in ICU. The secondary outcomes included 28-day all-cause mortality, length of ICU and hospital stay and the requirements of vasopressors or mechanical ventilation. A propensity score matching cohort was used to test the differences in primary and secondary outcomes between groups.

**Results:**

The results showed that vitamin D supplementation demonstrated a lower risk of sepsis (odd ratio 0.46; 95% CI 0.35–0.60; *P* < 0.001) and a lower risk of new mechanical ventilation requirement (odd ratio 0.70; 95% CI 0.53-0.92; *P* = 0.01), but no significant difference in the risk of 28-day mortality was observed (hazard ratio 1.02; 95% CI 0.77–1.35; *P* = 0.89). In the sensitive analysis, among the patients who suspected infection within 24 h before or after ICU admission, a lower risk of sepsis and a lower percentage of new mechanical ventilation also were detected in the vitamin D cohort.

**Conclusion:**

Vitamin D supplementation may have a positively prophylactic effect on sepsis in critically ill patients with suspected infection.

## Introduction

Sepsis is a clinical syndrome characterized by organ dysfunction due to a dysregulated host response to infection (1). Recently, although the introduction of international management guidelines and the implementation of the sepsis bundle strategy have significantly reduced the mortality of patients, sepsis still impacts millions of people annually and kills between one in three and one in six of them (2). In the United States, the medical cost of sepsis ranked highest among admissions for all diseases (3). Additionally, many survivors do not fully recover from the consequences of sepsis (4). Therefore, sepsis is a continuing threat to human life globally (5, 6). Due to the complicated pathogenesis and the heterogeneity of sepsis, no effective treatment was used despite numerous potential approaches for sepsis management. Research on new candidate therapies has gradually shifted the focus toward a new target: sepsis prophylaxis, which provides a promising method to fight against sepsis (7, 8).

Vitamin D is a fat-soluble nutrient element that plays an important role in the regulation of calcium and phosphate metabolism (9). It is also well-known for its effects on immunomodulation, infection prevention, and cardiovascular modulation (10, 11). The active form of vitamin D (1,25(OH)_2_D), induces macrophages and monocytes to produce endogenous antimicrobial cathelicidin LL-37 via VDR-RXR signaling, which exhibits an antimicrobial effect by destabilizing bacterial and fungal membranes. LL-37 also defenses against respiratory virus insult by directly disrupting viral envelopes and altering host cell viability (12, 13). Evidence from a clinical trial show that plasma LL-37 levels are significantly deficient in critically ill patients and vitamin D supplementation restores serum LL-37 levels in patients with sepsis (14, 15). Besides, 1,25(OH)_2_ protects the body from the overproduction of inflammatory cytokines by regulating innate immunity (12). Vitamin D and its metabolites also stabilize the endothelium through non-genomic actions to prevent vascular leakage (12, 13). The pathogen challenges and uncontrolled immune responses are the triggers of sepsis. Vitamin D with the effects of immunomodulation and anti-microbes may be an optimal agent to prevent sepsis.

Vitamin D deficiency is common in critically ill patients with severe infection and is strongly associated with increased mortality (14, 16). Vitamin D deficiency is an independent risk factor for sepsis, and higher 25-hydroxyvitamin D levels can reduce the incidence of sepsis (14, 17). A cohort study surveyed the association between vitamin D status and the incidence of sepsis in 81 patients with suspected infection, the results suggested that 79% of patients with suspected infection had vitamin D deficiency and its deficiency increased the risk of severe sepsis by more than 30% (18). Vitamin D deficiency is closely related to a higher risk of sepsis in critically ill patients. However, whether vitamin D supplementation is an effective strategy for sepsis prevention remains unclear. Therefore, we conducted a retrospective cohort study to investigate the effect of vitamin D supplementation on sepsis prevention in critically ill patients with suspected infection.

## Materials and methods

### Study design and study setting

We conducted a retrospective cohort study based on the Medical Information Mart for Intensive Care IV (MIMIC-IV 1.0 and MIMIC-IV 2.0). The database contains comprehensive data for more than 53,000 critically ill patients in intensive care units (ICU) from 2008 to 2019 at the Beth Israel Deaconess Medical Center, a tertiary care university hospital in Boston, USA (19). The authors are authorized to use this database. We followed the Strengthening the Reporting of Observational Studies in Epidemiology (STROBE) guidelines in this study (20). Since the database has been approved by the Institutional Review Board (IRB) of the Massachusetts Institute of Technology, the review from our IRB and the informed consent were exempted.

### Population and exposure

Adult patients aged 18–80 years old with suspected infection in the ICU were included. The suspected infection was defined as the administration of antibiotics in conjunction with a body-fluid culture (21). Patients will be excluded if they meet any following criterion: (1) the length of ICU stay (LOS) less than 24 h. (2) receiving vitamin D only after the diagnosis of sepsis or outside the ICU. (3) with a medication history of vitamin D two or more days prior to suspected infection. (4) with incomplete medication records of vitamin D. The recruited patients were divided into the vitamin D cohort or non-vitamin D cohort according to vitamin D administration status. For the patients with repeated ICU admission, only the ICU admission for the first suspected infection was included for analysis.

### Covariates and outcomes

The following variables collected within 24 h of ICU admission were extracted as the covariates: demographic characteristics, weight, admission type, comorbidities, vital signs, laboratory examination, sequential organ failure assessment score, Glasgow coma scale score, Simplified Acute Physiology Score II, and interventions including mechanical ventilation, vasopressors, continued renal replaced treatment and antibiotic. The first data were used for the variable with more than one data (22). After that, we also extracted the information of patients on the medication records of vitamin D in ICU, the occurrence of sepsis, LOS, survival status, and the requirement of vasopressors or mechanical ventilation. PostgreSQL 13.0 was used for data extraction.

The primary outcomes were occurrence of sepsis in ICU. The Sepsis-3 criteria were used for the diagnosis of sepsis (1, 21). The secondary outcomes included 28-day all-cause mortality, length of ICU and hospital stay, duration of vasopressors or mechanical ventilation, and new requirement of vasopressors or mechanical ventilation (defined as the first mechanical ventilation or vasopressors administration occurred after 24 h of ICU admission).

### Statistical analysis

Categorical variables were reported as frequencies and percentages, and the differences between groups were compared using chi-square test or logistics regression. Continuous variables were presented as medians and interquartile (IQR), and the corresponding differences between groups were compared by Wilcox signed-rank test. A Cox regression model was used to estimate the association between 28-day all-cause mortality and vitamin D administration. Missing values at random were imputed by a random forest model. As the laboratory variables for B-type natriuretic peptide, hemoglobin, serum sodium, serum potassium, and serum albumin were missing in more than half of the cohort, these variables were transformed as binary variables based on their missing data (22) (Supplementary Figure 1).

A logistics regression model was employed to calculate the propensity score of each patient for vitamin D administration. Then, a 1:1 nearest propensity score matching (PSM) model with a caliper of 0.05 was applied for the causal effect of vitamin D on primary and secondary outcomes. To examine the balance of baseline characteristics, the standardized mean difference (SMD) of each variable between the two cohorts was calculated. SMD commonly be applied to evaluate baseline balance between groups before and after PSM model, and a value less than 0.1 of SMD is considered a balance (23). After that, the effectiveness of vitamin D in the primary outcomes was estimated by a conditional logistic regression model. Moreover, a multivariate regression model, a stabilized inverse probability of treatment weighting model (SIPTW), and a double robust model were also applied to evaluate the robustness of our results. For the SIPTW model, the stabilized weights were calculated to generate a weighted cohort, as described by Xu et al. (24). Then, the effectiveness of the vitamin D in the primary outcomes was estimated based on this weighted cohort by the weighted logistic regression. For the doubly robust model, a SIPTW model combined with a multivariable logistic regression with all baseline variables was used to estimate the effectiveness of vitamin D (22, 25). Furthermore, we also conducted a sensitivity analysis only included patients with a suspected infection within 24 h before or after ICU admission to assess the robustness of the results. The subgroup analyses of sepsis risk in ICU stratified by age, gender, liver disease, malignancy, diabetes, chronic heart disease to assess the interaction between vitamin D administration and these stratified groups. Finally, to assess the potential effect of unmeasured confounding, the E-value was calculated when an outcome was statistically significant, which represents to the minimum strength of the effect of the unmeasured confounders on the treatment and on the outcome (26, 27).

All analyses were performed using the R 4.1.0 software (R Foundation for Statistical Computing, Vienna, Austria), and *P*-values less than 0.05 were considered statistically significant. All comparisons between the groups used the non-vitamin D cohort as a reference.

## Results

**FIGURE 1:**
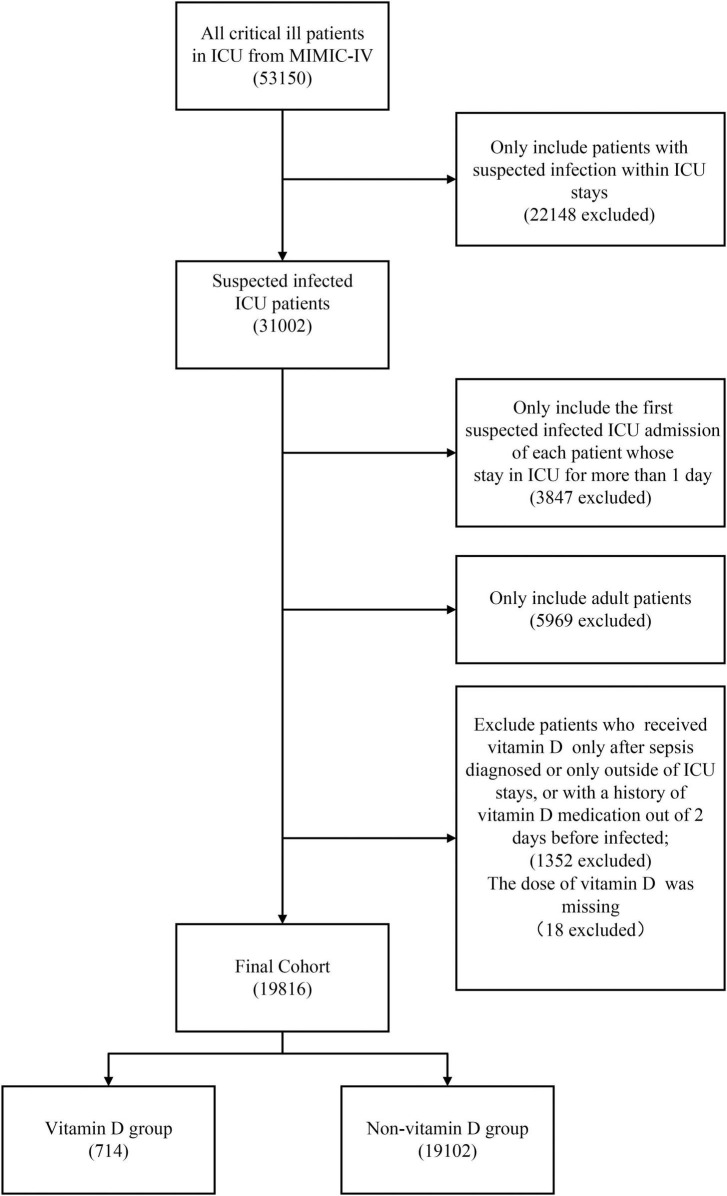
The flowchart on the selection of the study population.

Figure 1 shows the screening of the study population. Overall, a total of 31,002 patients with suspected infection in ICU were identified. After excluding the patients by the exclusion criteria, a total of 19,816 patients were included in our cohorts. Of these, 3.6% of the patients were in the vitamin D cohort in which the median duration and the median maximum daily dose of vitamin D administration were 6.42 days (IQR, 3.42–10.67) and 1,000 IU (IQR, 800–1,000), respectively. The detailed characteristics of vitamin D administration are presented in Supplementary Figures 2, 3. The patients in the vitamin D cohort were older and more likely to be female and had a higher presence of comorbidity of congestive heart failure, diabetes and chronic kidney disease compared with the non-vitamin D cohort. Inversely, a higher proportion of the patients in the non-vitamin D cohort received mechanical ventilation and vasopressor during the first 24 h of their ICU stay. After PSM, all the baseline characteristics between the groups were balanced (Table 1).

**TABLE 1:** The clinical characteristics of original cohort and propensity score matching cohort.

Covariate	Original cohort			Propensity score matching cohort		
	No Vitamin D	Vitamin D	SMD	No Vitamin D	Vitamin D	SMD
No. of patients	19,102	714		713	713	
Age, (median [IQR]), year	62.50 [52.42, 70.85]	67.54 [59.67, 73.65]	0.407	68.07 [60.00, 74.61]	67.48 [59.67, 73.65]	0.019
Gender, No. (%), male	11,599 (60.7)	311 (43.6)	0.349	333 (46.7)	311 (43.6)	0.062
Ethnicity, (%), white	12,516 (65.5)	516 (72.3)	0.146	538 (75.5)	515 (72.2)	0.073
Weight, (median [IQR]), kg	82.80 [69.80, 98.30]	80.00 [65.40, 95.00]	0.126	81.40 [67.40, 94.55]	80.00 [65.30, 95.00]	0.005
Emergency admission (%)	11,073 (58.0)	417 (58.4)	0.009	428 (60.0)	416 (58.3)	0.034
**Comorbidities, No. (%)**						
Myocardial infarct	2,935 (15.4)	108 (15.1)	0.007	114 (16.0)	108 (15.1)	0.023
Congestive heart failure	4,332 (22.7)	225 (31.5)	0.2	234 (32.8)	224 (31.4)	0.03
Cerebrovascular disease	2,638 (13.8)	83 (11.6)	0.066	94 (13.2)	83 (11.6)	0.047
COPD	938 (4.9)	54 (7.6)	0.11	46 (6.5)	54 (7.6)	0.044
Diabetes	5,537 (29.0)	275 (38.5)	0.203	295 (41.4)	274 (38.4)	0.06
Liver disease	3,090 (16.2)	93 (13.0)	0.089	82 (11.5)	93 (13.0)	0.047
CKD	1,896 (9.9)	98 (13.7)	0.118	111 (15.6)	98 (13.7)	0.052
Malignant cancer	2,618 (13.7)	101 (14.1)	0.013	110 (15.4)	101 (14.2)	0.036
**Interventions at ICU addmission, No. (%)**						
Mechanical ventilation	9,480 (49.6)	205 (28.7)	0.439	191 (26.8)	205 (28.8)	0.044
Vasopressor	7,944 (41.6)	211 (29.6)	0.253	208 (29.2)	211 (29.6)	0.009
CRRT	354 (1.9)	9 (1.3)	0.048	13 (1.8)	9 (1.3)	0.046
**Vital signs**						
Heart rate (median [IQR])	88.00 [77.00, 103.00]	86.00 [76.00, 102.00]	0.051	87.00 [76.00, 104.00]	86.00 [76.00, 102.00]	0.045
Resp rate (median [IQR])	18.00 [15.00, 22.00]	18.00 [15.00, 23.00]	0.07	18.00 [15.00, 23.00]	18.00 [15.00, 23.00]	0.019
MAP (median [IQR])	82.00 [71.00, 94.00]	80.00 [69.00, 91.50]	0.11	80.00 [68.00, 91.00]	80.00 [69.00, 92.00]	0.051
Temperature,°C, (median [IQR])	36.72 [36.39, 37.11]	36.72 [36.44, 37.06]	0.013	36.78 [36.44, 37.11]	36.72 [36.44, 37.06]	0.07
**Laboratory examination**						
Hemoglobin (tested), No. (%)	7,002 (36.8)	222 (31.3)	0.117	214 (30.0)	222 (31.1)	0.024
Platelet, (median [IQR]), K/μL	188.00 [132.00, 257.00]	196.00 [133.50, 266.00]	0.069	196.00 [141.00, 268.00]	196.00 [134.00, 266.00]	0.016
Creatinine, (median [IQR]), ng/mL,	0.90 [0.70, 1.40]	0.90 [0.70, 1.40]	0.006	0.90 [0.70, 1.40]	0.90 [0.70, 1.40]	0.022
WBC, (median [IQR]), K/μL	11.50 [8.00, 15.90]	10.00 [7.25, 14.25]	0.149	10.50 [7.60, 14.10]	10.00 [7.30, 14.20]	0.017
pH (median [IQR])	7.38 [7.32, 7.43]	7.39 [7.33, 7.44]	0.169	7.39 [7.34, 7.44]	7.39 [7.34, 7.44]	0.019
Sodium (tested), No. (%)	7,227 (38.0)	221 (31.1)	0.145	214 (30.0)	221 (31.0)	0.021
Calcium, (median [IQR]), mg/dL	8.20 [7.70, 8.80]	8.40 [7.90, 8.90]	0.214	8.40 [7.90, 8.90]	8.40 [7.90, 8.90]	0.098
Albumin (tested) (%)	6614 (34.8)	198 (27.9)	0.149	203 (28.5)	199 (27.9)	0.012
BUN, (median [IQR]), mg/dL	18.00 [12.00, 28.00]	18.00 [12.00, 29.00]	0.015	19.00 [13.00, 29.00]	18.00 [12.00, 29.00]	0.035
Bicarbonate, (median [IQR]), mEq/L	23.00 [20.00, 25.00]	24.00 [21.00, 26.00]	0.241	23.00 [21.00, 26.00]	24.00 [21.00, 26.00]	0.046
BNP (tested), No. (%)	795 (4.2)	43 (6.1)	0.085	32 (4.5)	43 (6.0)	0.069
PCO2, (median [IQR]), mmHg	41.00 [36.00, 47.00]	41.00 [36.00, 48.00]	0.135	42.00 [36.00, 47.00]	41.00 [35.00, 48.00]	0.028
Potassium (tested), No. (%)	8375 (44.0)	246 (34.6)	0.193	229 (32.1)	246 (34.5)	0.051
PO2, (median [IQR]), mmHg	140.00 [71.00, 312.00]	112.00 [61.00, 308.00]	0.067	98.00 [59.00, 217.00]	98.00 [57.00, 221.00]	0.027
Lactate, (median [IQR]), mmol/L	1.60 [1.20, 2.50]	1.50 [1.10, 2.20]	0.201	1.50 [1.10, 2.10]	1.50 [1.10, 2.10]	0.005
SOFA (median [IQR])	5.00 [3.00, 8.00]	4.00 [2.00, 7.00]	0.344	4.00 [3.00, 7.00]	4.00 [2.00, 7.00]	0.037
GCS (median [IQR])	14.00 [10.00, 15.00]	14.00 [13.00, 15.00]	0.254	14.00 [12.00, 15.00]	14.00 [13.00, 15.00]	0.029
SAPSII (median [IQR])	34.00 [26.00, 44.00]	32.00 [25.00, 42.00]	0.124	34.00 [27.00, 40.00]	32.00 [25.00, 42.00]	0.046

BUN, blood urea nitrogen; BNP, B-type natriuretic peptide; COPD, chronic obstructive pulmonary disease; CKD, chronic kidney disease; CRRT, continuous renal replaced treatment; GCS, Glasgow coma scale; IQR, interquartiles; MAP, mean arterial pressure; SAPSII, simplified acute physiology score II; SOFA, sequential organ failure assessment; SMD, standardized mean differences; WBC, White blood cell; In the comparation of baseline characteristics, a value less than 0.1 of SMD is considered as a balance.

### Primary and secondary outcomes

**FIGURE 2:**
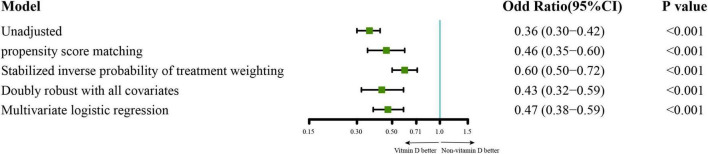
The analysis of sepsis risk with five different methods.

For the primary outcomes, the incidence of sepsis in the vitamin D cohort and non-vitamin D cohort were 70.4 vs. 83.7%. The analysis of PSM demonstrate a lower risk of sepsis in patients with vitamin D administration, and the adjusted odd risk was 0.46 (95% CI 0.35–0.60; *P* < 0.001; *E*-value = 2.31). This effect is also statistically significant in the multivariable logistic regression, the SIPTW model, and the doubly robust model. The adjusted odd risk of these three models were 0.47 (95% CI 0.38–0.59; *P* < 0.001; *E*-value = 2.28), 0.60 (95% CI 0.50–0.72; *P* < 0.001; *E*-value = 1.90) and 0.43 (95% CI 0.32–0.59; *P* < 0.001; *E*-value = 2.42), respectively (Figure 2). In the analysis of the secondary outcomes with PSM cohort, no significant difference in the risk of 28-day all-cause mortality was observed in PSM cohort (hazard ratio 1.02; 95% CI 0.77–1.35; *P* = 0.89; Supplementary Figure 4). we also estimated the effect of vitamin D on clinically meaningful outcomes including length of ICU and hospital stays, duration of mechanical ventilation or vasopressors and incidence of new mechanical ventilation or vasopressors administration during ICU stays. The results showed that patients in vitamin D cohort had a lower risk of new mechanical ventilation than that in non-vitamin D cohort (Odd ratio 0.70; 95%CI 0.53-0.92; *P* = 0.01; *E*-value = 1.67). There were no significant differences in the other secondary outcomes (Table 2).

**TABLE 2:** Primary and secondary outcomes analysis with propensity score matching cohort.

Outcomes	Vitamin D (*n* = 713)	No vitamin D (*n* = 713)	Treatment effect (95% CI)^d^	*P*-value
**Primary outcome**				
Sepsis risk in ICU	502 (70.4), *n* = 713	597 (83.7), *n* = 713	0.46 (0.35-0.60)	< 0.001
**Secondary outcomes**				
28-all caused mortality, n (%)	101 (14.2), *n* = 713	98 (13.7), *n* = 713	1.02 (0.77–1.35)	0.89
The length of ICU stays, d ^a^	2.78 (1.57-4.85), *n* = 713	2.37 (1.54-4.15), *n* = 713	0.06 (−0.19-0.31)	0.64
The length of hospital, d ^a^	8.00 (5.31-12.79), *n* = 713	7.94 (5.14-13.99), *n* = 713	−0.43 (−1.13, 0.25)	0.23
The duration of mechanical ventilation, d ^b^	0.76 (0.23-3.80), *n* = 326	0.75 (0.25-2.51), *n* = 350	0.04 (−0.05, 0.16)	0.34
The incidence of new mechanical ventilation during ICU stays, (n)% ^c^	121 (23.8), *n* = 508	161 (30.8), *n* = 522	0.70 (0.53-0.92)	0.01
The duration of vasopressors, d ^b^	0.87 (0.20-2.56), *n* = 260	0.65 (0.23-2.43), *n* = 253	0.03 (−0.09,0.19)	0.61
The incidence of new vasopressors administration during ICU stays,% ^c^	49 (9.8%), *n* = 502	45 (8.9%), *n* = 505	1.11 (0.72-1.70)	0.64

^a^The significances between was calculated by paired Wilcoxon signed rank test because of the paired design. ^b^Only including the patients with mechanical ventilation or vasopressors. ^c^new vasopressors administration or new mechanical ventilation was defined as the first mechanical ventilation or vasopressors administration after 24 h of ICU admission. ^d^Hazard ratio was reported for risk of 28-day mortality, odd ratios were reported for categorical variables, and differences between groups were reported for continuous variables.

### Subgroup and sensitivity analysis

**FIGURE 3:**
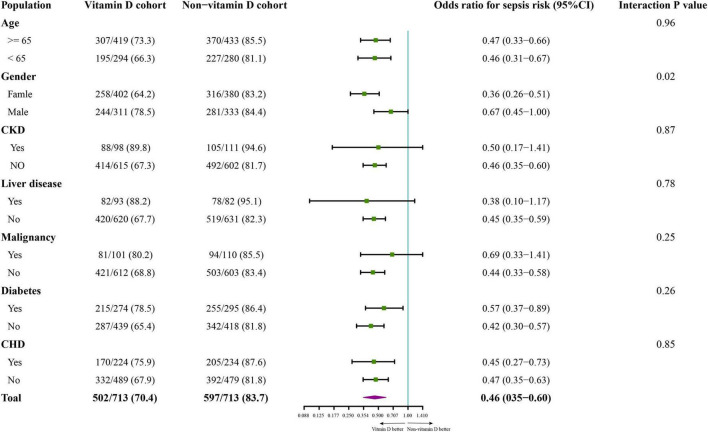
The subgroup analysis on incidence of sepsis in the propensity score matching cohort. The variables of subgroup analysis, the number of patients with sepsis (numerator), the number of each subgroup population (denominator), the odd ratios for incidence of sepsis, 95% CI in each comparison, and the *p*-value of interaction between the cohorts (vitamin D cohort or non-vitamin D cohort) and the grouped variables are shown in the forest plot. CKD, chronic kidney disease; CHD, chronic heart disease.

The association between vitamin D and the covariables that may influence the effect of vitamin D was evaluated by subgroup analysis for sepsis risk (28) and a significant interaction between gender and vitamin D supplementation was detected (*P* = 0.02). Specifically, a stronger prophylactic effect of vitamin D was detected in female patients (Odd ratio 0.36; 95% CI 0.26-0.51) than male patients (Odd ratio 0.67; 95% CI 0.45-1.00). No significant interaction between vitamin D supplementation and the other factors was observed (Figure 3). Additionally, in the sensitivity analysis of 1,394 patients who were suspected infected within 24 h before or after ICU admission, the risk of sepsis (Odd ratio 0.59; 95% CI 0.46-0.76; *P* < 0.001) and the risk of new mechanical ventilation (Odd ratio 0.69; 95% CI 0.52-0.91; *P* = 0.01) was also decreased in vitamin D cohort (Supplementary Tables 1, 2).

## Discussion

Sepsis is associated with poor clinical outcomes, including high mortality, and high medical costs. Prevention of sepsis is crucial for critically ill patients (9). Vitamin D deficiency is associated with a higher predisposition to infection (29). Furthermore, a large cohort study on US adults showed that vitamin D deficiency was a strong independent risk of sepsis (30). As the deficiency of vitamin D has been observed widely, vitamin D administration should be considered for critically ill patients, especially those with infection (11). Although in a multicenter, double-blind, randomized, placebo-controlled trial, no benefit of vitamin D was detected for treating moderate to severe COVID-19 whose pathology is similar to that of sepsis. The authors believed that further studies should be conducted to assess the preventative role of vitamin D (31). To our knowledge, our study is the first study with a large sample size to assess the effect of vitamin D on sepsis prophylaxis in critically ill patients with suspected infection.

In the PSM cohort, vitamin D supplementation significantly decreased the risk of sepsis in critically ill patients with suspected infection and the same finding was also observed in the other three models. In the study, as many covariates as possible were included to adjust the result. Meanwhile, the *E*-value was 2.31, which means that only residual confounding with an odd risk of at least 2.31 could the observe result negate if there exists an unmeasured confounding. Additionally, in the sensitivity analysis, we included the patients suspected infected within 24 h before or after ICU admission only. In this population, the baseline characteristics better reflected the status of the patients at the time of suspected infection and the results also support the effect of vitamin D on reducing the risk of sepsis. Therefore, we believed that the association between vitamin D supplementation and a lower risk of sepsis was robust. Sepsis has its “golden time” and the early recognition and appropriate intervention improve outcomes (5). In the vitamin D cohort, we observed that most patients were given the first vitamin D as soon as the suspected infection (Supplementary Figure 2A), which was beneficial to the infection control and the immunomodulation. However, the administration of vitamin D did not improve the 28-survival. In our analysis, since lacking of available data, the endogenous vitamin D levels of the patients were not considered, and the median daily dose of vitamin D (median 800 IU; IQR 800-1,000) in this study was much lower than that of the randomized controlled trial that showed an improved survival rate (32). The administration of high-dose vitamin D3 has been proven to be safe and restores the level of 25-hydroxyvitamin D in critical patients with vitamin D-deficient (31–33). Therefore, a further clinical trial should be conducted to determine the effects of high-dose vitamin D on mortality and other non-fatal outcomes in critically ill patients with suspected infection, especially in those with vitamin D-deficient. Noteworthily, vitamin D supplementation decreased the requirement for new mechanical ventilation by 7% in the analysis of secondary outcomes. In a multicenter randomized controlled trial of vitamin D in the patients with moderate to severe COVID-19, vitamin D administration reduced the mechanical ventilation requirement by 6.8%. However, the power of the sample size in that trial was inadequate to detect this clinically meaningful difference (–6.8 [95% CI, –15.1–1.2], *P* = 0.09) (31). In our study, we investigated the difference in a larger sample size and the result indicated that vitamin D supplementation probably decreased the new mechanical ventilation requirement in critically ill patients with suspected infection. In our study, new mechanical ventilation means that the need for mechanical ventilation arose during ICU stay rather than at the time of ICU admission, inflecting the deterioration of respiratory function in ICU stay. The lower requirement for new mechanical ventilation in vitamin D cohort suggests the positive role of vitamin D in preventing respiratory dysfunction. Therefore, the mechanical ventilation requirement should be considered a main outcome in the further randomized controlled trials of vitamin D supplementation in critically ill patients.

In this study, we investigated the effect of vitamin D in critically ill patients with suspected infection in a large sample size. Meanwhile, we excluded the patients who had a history of vitamin D supplements before the suspected infection, which allowed us to estimate the immediate effects of vitamin D in the acute phase of infection rather than its cumulative effects. Our findings showed a positive effect of vitamin D in critically ill patients with suspected infection, providing a target population for a further randomized controlled trial. However, several limitations are unavoidable in our study. First, as we judged the vitamin D administration by the record of prescription, the medication compliance was unclear. Second, the administration dose of vitamin D was flexible in several patients, which makes it impossible for further analysis of the effect of vitamin D at a fixed-dose. Third, some clinically meaningful outcomes such as medical cost, could not be evaluated due to the unavailability of data. Forth, the data on the vitamin D levels of the included patients were unavailable since few patients underwent vitamin D testing. In future prospective studies, it will be necessary to examine vitamin D levels in critically ill patients with suspected infection to determine the association between restoring vitamin D levels through vitamin D supplementation and the risk of sepsis. Moreover, as a retrospective study, potential unknown bias or confounding perhaps exists.

## Conclusion

The cohort study suggested that vitamin D supplementation may have a positively prophylactic effect on sepsis in critically ill patients with suspected infection. The effects of vitamin D on this target population should be determined by a further randomized controlled trial.

## Data availability statement

All raw data supporting the outcomes of this article can be obtained from the MIMIC-IV database (https://mimic.physionet.org/) and the code of data extraction are available from the corresponding author on reasonable request by sending to ZLiu, zhguoliu@163.com.

## Ethics statement

The studies involving human participants were reviewed and approved by Beth Israel Deaconess Medical Center (Boston, MA) and Massachusetts Institute of Technology (Cambridge, MA), and consent was obtained for the original data collection. The authors of the manuscript have been authorized to utilize the database. Written informed consent for participation was not required for this study in accordance with the national legislation and the institutional requirements.

## Author contributions

ZLiu, PC, and YL designed the study and take responsibility for the integrity of the data and the accuracy of the data analysis. JG, MS, and ZLia conducted data collection and analysis. JG, MS, and ZLiu wrote the manuscript. ZLiu, PC, YL, and JG interpreted the results. ZLia, SY, and LZ checked the data and revised the results. SY, MZ, and RL reviewed and edited the manuscript. All authors approved this manuscript.
