# Supplementary material for: Potential benefits of vitamin D for sepsis prophylaxis in critical ill patients
Source: Front Nutr. 2023 Apr 4;10:1073894. doi: 10.3389/fnut.2023.1073894 (PMC10110989; doi:10.3389/fnut.2023.1073894)
Supplement: Supplementary file 1 [file Data_Sheet_1.docx]

**Supplemental files**

**Potential benefits of vitamin D for sepsis prophylaxis in critical ill patients**

**Contents**

1. **Figure S1. The histogram of the proportion of missing value in the original cohort.……………………………………………………..……2**
2. **Figure S2. The Raincloud plots of the characteristics of vitamin D administration……...........................................................................3**
3. **Figure S3. The timeline characteristic of the study……………….4**
4. **Figure S4.** **The Kaplan–Meier survival curve of mortality during the 28 day follow-up in the PSM cohort………………………………...5**
5. **Table S1. The baseline characteristic of the sensitivity analysis in patients who suspected infected within 24h before or after ICU admission……….6**
6. **Table S2. The sensitivity analysis of primary and secondary outcomes with propensity score matching cohort……………………..…….…..8**

**
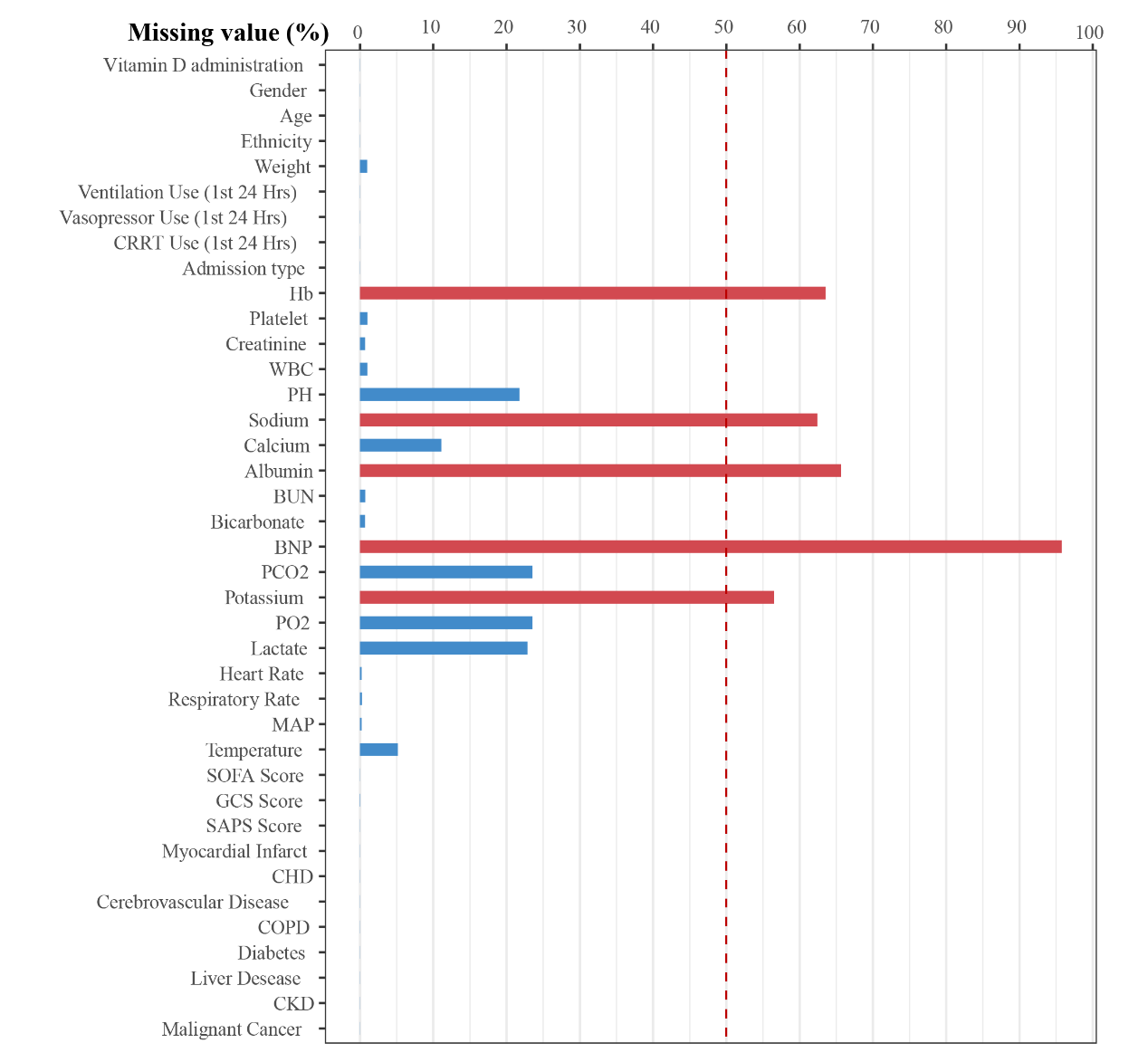
**

**Figure S1.** **The histogram of the proportion of missing value in the original cohort.** The figure shows the proportion of miss value of baseline variables. The baseline variables with more than 50% missing are transformed as binary variables in the analysis of baseline characteristics.

**
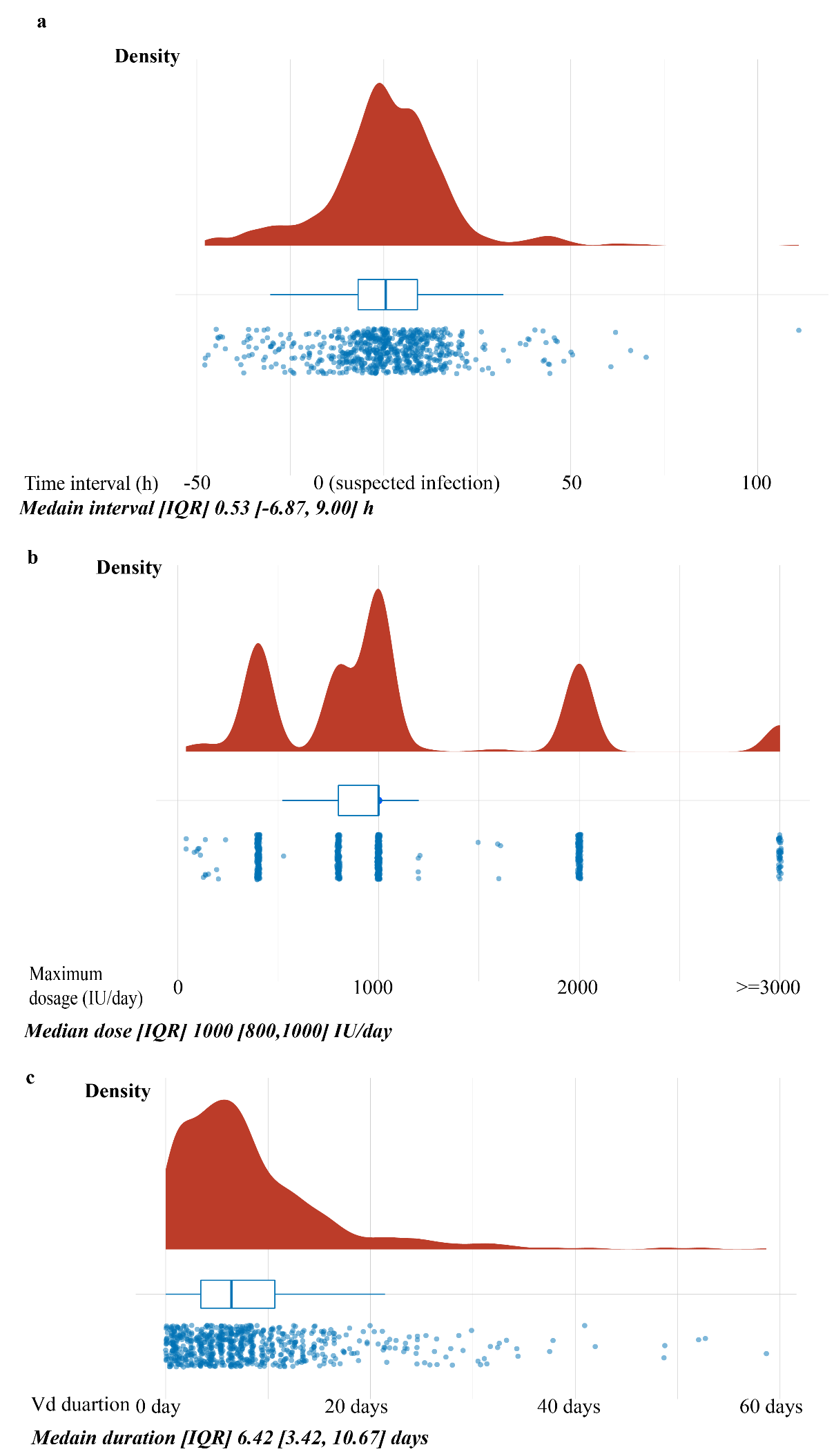
**

**Figure S2. The Raincloud plots of the characteristics of vitamin D administration.** Panel A shows the time interval from the suspected infection to the first vitamin D supplementation. The density plot (red) presents the characteristics of the time interval. The box-plot and the dot-plot (blue) present the time interval of each patient in vitamin D cohort. Panel B shows the daily dose of vitamin D supplementation. The density plot (red) presents the characteristics of the maximum daily dose of vitamin D supplementation. The box-plot and the dot-plot (blue) present the maximum daily dose of vitamin D supplementation in the vitamin D cohort. Panel C shows the durations of vitamin D supplementation. The density plot (red) presents the characteristics of the durations of vitamin D supplementation. The box-plot and the dot-plot (blue) present the durations of vitamin D supplementation in the vitamin D cohort.

**
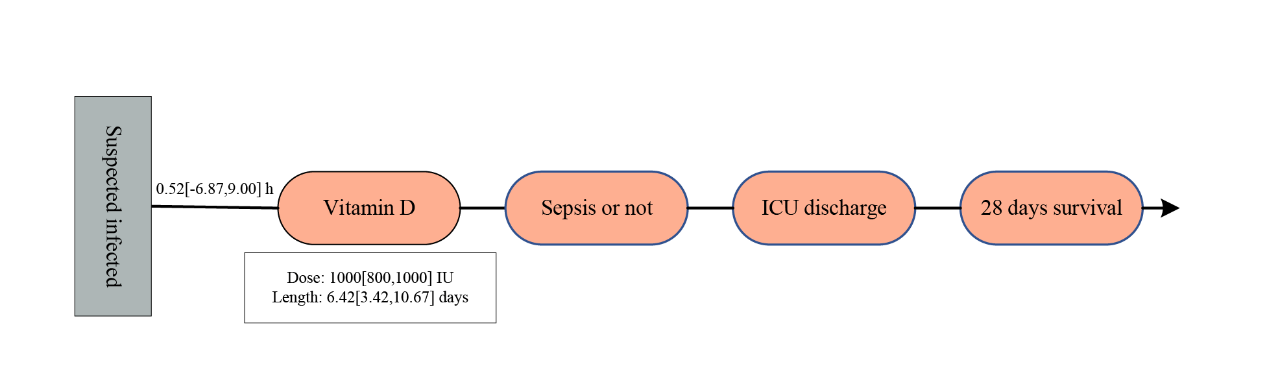
**

**Figure S3.** **The timeline characteristic of the study.**

**
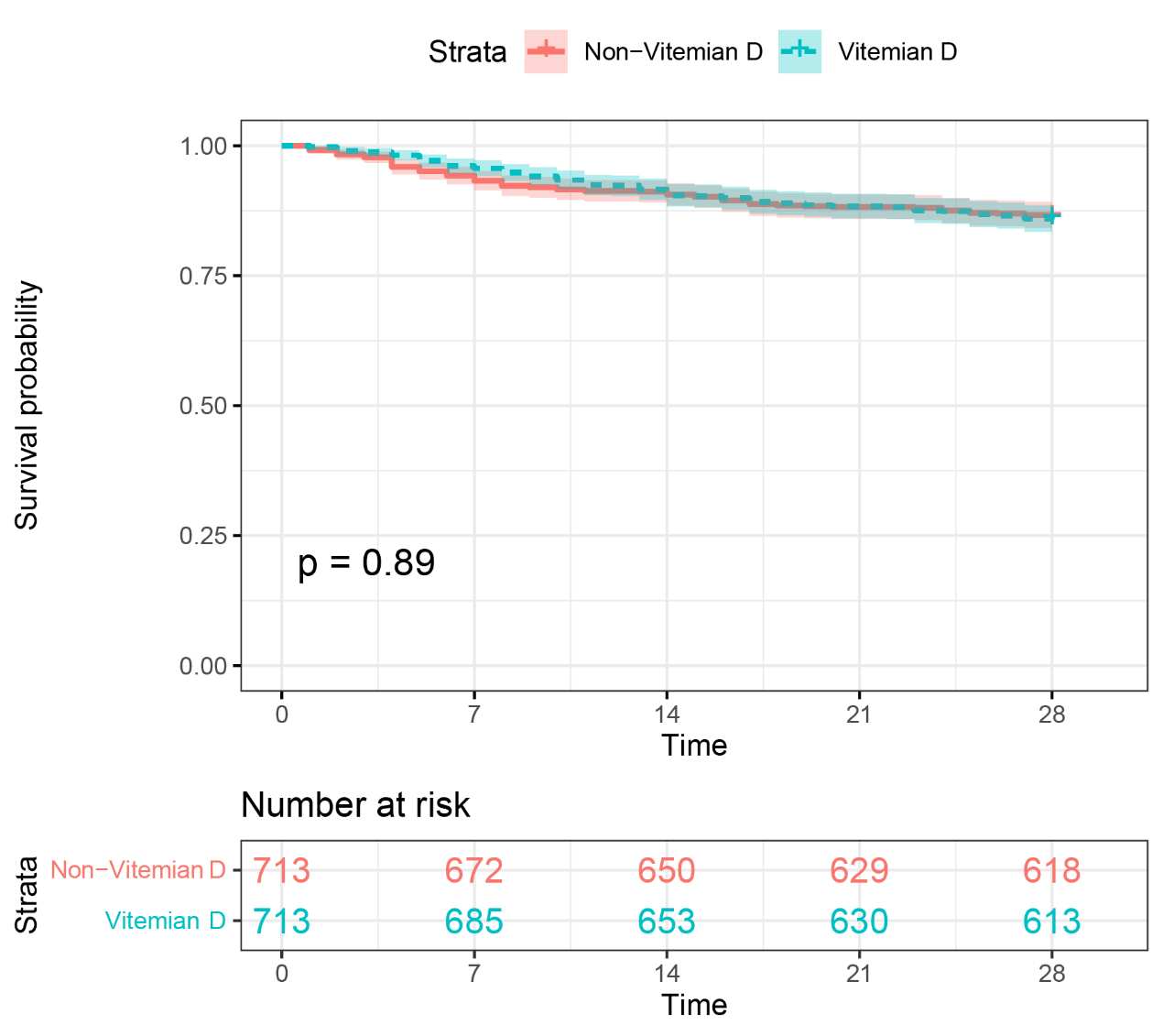
**

**Figure S4. The Kaplan–Meier survival curve of mortality during the 28 days follow-up in the PSM cohort.** The information of 28-day survival was extracted from the patient table in MIMIC 2.0. The difference of 28-day survival between groups were detected by log rank test.

**Table S1.** **The baseline characteristic of the sensitive analysis population who** **suspected infected within 24h before or after ICU admission.**

| **Covariate** | **Original cohort** | | | **Propensity score matching cohort** | | |
| --- | --- | --- | --- | --- | --- | --- |
|  | **No Vitamin D** | **Vitamin D** | **SMD** | **No Vitamin D** | **Vitamin D** | **SMD** |
| n | 18062 | 700 |  | 697 | 697 |  |
| Age, (median [IQR]), yr | 62.49 [52.42, 70.83] | 67.61 [59.90, 73.72] | 0.424 | 68.25 [60.12, 74.29] | 67.59 [59.77, 73.67] | 0.037 |
| Gender, No. (%), male | 10984 (60.8) | 305 (43.6) | 0.35 | 310 (44.5) | 305 (43.8) | 0.014 |
| Ethnicity, No. (%), white | 11881 (65.8) | 508 (72.6) | 0.147 | 504 (72.3) | 506 (72.6) | 0.006 |
| Weight, (median [IQR]), kg | 82.80 [69.90, 98.33] | 79.90 [65.30, 94.62] | 0.136 | 81.00 [68.00, 95.50] | 80.10 [65.40, 95.00] | 0.024 |
| Emergency admission, No. (%) | 10374 (57.4) | 408 (58.3) | 0.017 | 394 (56.5) | 405 (58.2) | 0.032 |
| **Comorbidities, No. (%)** |  |  |  |  |  |  |
| Myocardial infarct | 2775 (15.4) | 103 (14.7) | 0.018 | 112 (16.1) | 103 (14.8) | 0.036 |
| Congestive heart failure | 4098 (22.7) | 220 (31.4) | 0.198 | 230 (33.0) | 217 (31.1) | 0.040 |
| Cerebrovascular disease | 2276 (12.6) | 79 (11.3) | 0.041 | 66 (9.5) | 79 (11.3) | 0.061 |
| COPD | 854 (4.7) | 52 (7.4) | 0.113 | 47 (6.7) | 52 (7.5) | 0.028 |
| Diabetes | 5278 (29.2) | 269 (38.4) | 0.196 | 281 (40.3) | 267 (38.3) | 0.041 |
| Liver disease | 2978 (16.5) | 93 (13.3) | 0.09 | 77 (11.0) | 92 (13.2) | 0.066 |
| CKD | 1821 (10.1) | 96 (13.7) | 0.112 | 101 (14.5) | 96 (13.8) | 0.021 |
| Malignant cancer | 2503 (13.9) | 101 (14.4) | 0.016 | 102 (14.6) | 101 (14.5) | 0.004 |
| **Interventions at ICU admission, No. (%)** |  |  |  |  |  |  |
| Mechanical ventilation | 8937 (49.5) | 201 (28.7) | 0.436 | 208 (29.8) | 201 (28.8) | 0.022 |
| Vasopressor | 7613 (42.1) | 211 (30.1) | 0.252 | 234 (33.6) | 211 (30.3) | 0.071 |
| CRRT | 341 (1.9) | 9 (1.3) | 0.048 | 5 (0.7) | 9 (1.3) | 0.058 |
| **Vital signs** |  |  |  |  |  |  |
| Heart rate (median [IQR]) | 88.00 [78.00, 103.00] | 87.00 [76.00, 102.00] | 0.053 | 86.00 [76.00, 101.00] | 86.00 [76.00, 102.00] | 0.047 |
| Resp rate (median [IQR]) | 18.00 [15.00, 22.00] | 18.00 [15.00, 23.00] | 0.062 | 18.00 [15.00, 23.00] | 18.00 [15.00, 23.00] | 0.059 |
| MAP (median [IQR]) | 81.00 [71.00, 93.00] | 80.00 [69.00, 91.00] | 0.098 | 80.00 [70.00, 91.00] | 80.00 [69.00, 92.00] | 0.039 |
| Temperature, (median [IQR]), °C | 36.72 [36.39, 37.11] | 36.72 [36.44, 37.06] | 0.010 | 36.70 [36.39, 37.06] | 36.72 [36.44, 37.06] | 0.083 |
| **Laboratory examination** |  |  |  |  |  |  |
| Hemoglobin(tested), No. (%) | 6724 (37.4) | 222 (31.9) | 0.116 | 231 (33.1) | 223 (32.0) | 0.024 |
| Platelet, (median [IQR]), K/μL | 188.00 [131.00, 258.00] | 195.00 [134.00, 267.00] | 0.073 | 199.00 [146.00, 267.00] | 196.00 [134.00, 267.00] | 0.019 |
| Creatinine, (median [IQR]), ng/mL | 0.90 [0.70, 1.40] | 0.90 [0.70, 1.40] | 0.014 | 0.90 [0.70, 1.30] | 0.90 [0.70, 1.40] | 0.039 |
| WBC, (median [IQR]), K/μL | 11.60 [8.00, 16.00] | 10.00 [7.30, 14.30] | 0.147 | 10.80 [7.20, 14.70] | 10.10 [7.30, 14.40] | 0.007 |
| pH (median [IQR]) | 7.38 [7.32, 7.43] | 7.39 [7.33, 7.44] | 0.168 | 7.39 [7.34, 7.44] | 7.39 [7.34, 7.44] | 0.015 |
| Sodium(tested), No. (%) | 6943 (38.6) | 220 (31.6) | 0.147 | 233 (33.4) | 221 (31.7) | 0.037 |
| Calcium, (median [IQR]), mg/dL | 8.20 [7.70, 8.70] | 8.40 [7.90, 8.90] | 0.216 | 8.30 [7.80, 8.80] | 8.40 [7.90, 8.90] | 0.094 |
| Albumin (tested), No. (%) | 6294 (35.0) | 195 (28.0) | 0.151 | 206 (29.6) | 196 (28.1) | 0.032 |
| BUN, (median [IQR]), mg/dL | 18.00 [13.00, 28.00] | 18.00 [12.00, 29.25] | 0.001 | 18.00 [13.00, 29.00] | 18.00 [12.00, 29.00] | 0.034 |
| Bicarbonate, mEq/L, (median [IQR]) | 23.00 [20.00, 25.00] | 24.00 [21.00, 26.00] | 0.242 | 24.00 [21.00, 26.00] | 24.00 [21.00, 26.00] | 0.006 |
| BNP (tested), No. (%) | 767 (4.3) | 41 (5.9) | 0.074 | 55 (7.9) | 41 (5.9) | 0.079 |
| PCO2, (median [IQR]), mmHg | 41.00 [36.00, 47.00] | 41.00 [36.00, 48.00] | 0.133 | 41.00 [36.00, 48.00] | 41.00 [36.00, 48.00] | 0.010 |
| Potassium (tested), No. (%) | 8013 (44.6) | 244 (35.1) | 0.195 | 251 (36.0) | 245 (35.2) | 0.018 |
| PO2, (median [IQR]), mmHg | 141.00 [70.00, 318.00] | 112.00 [61.00, 311.50] | 0.072 | 104.00 [62.00, 249.00] | 97.00 [57.00, 234.00] | 0.052 |
| Lactate, (median [IQR]), mmol/L, | 1.60 [1.20, 2.50] | 1.50 [1.10, 2.20] | 0.198 | 1.50 [1.10, 2.20] | 1.50 [1.10, 2.20] | 0.025 |
| SOFA (median [IQR]) | 5.00 [3.00, 8.00] | 4.00 [2.00, 7.00] | 0.343 | 4.00 [2.00, 6.00] | 4.00 [2.00, 7.00] | 0.018 |
| GCS (median [IQR]) | 14.00 [10.00, 15.00] | 14.00 [13.00, 15.00] | 0.233 | 14.00 [13.00, 15.00] | 14.00 [13.00, 15.00] | 0.004 |
| SAPSII (median [IQR]) | 34.00 [26.00, 44.00] | 33.00 [25.00, 42.00] | 0.122 | 33.00 [26.00, 41.00] | 33.00 [26.00, 41.00] | 0.013 |

BUN = Blood urea nitrogen; BNP = B-type natriuretic peptide; COPD = chronic obstructive pulmonary disease; CKD = chronic kidney disease; CRRT = continuous renal replaced treatment; GCS = Glasgow coma scale; IQR = interquartiles; MAP = mean arterial pressure; SAPSII = simplified acute physiology score II; SOFA = sequential organ failure assessment; SMD = standardized mean differences; WBC = White blood cell; In the comparation of baseline characteristics, a value less than 0.1 of SMD is considered as a balance.

**5. Table S2. The sensitivity analysis of primary and secondary outcomes with propensity score matching cohort**

| **Outcomes** | **Vitamin D (n=697)** | **No vitamin D (n=697)** | **Treatment effect**  **(95% CI) ^d^** | **P value** |
| --- | --- | --- | --- | --- |
| **Primary outcome** |  |  |  |  |
| The incidence of sepsis in hospital, n (%) | 490 (70.3), n=697 | 557 (79.9), n=697 | 0.59 (0.46-0.76) | **<0.001** |
| **Secondary outcomes** |  |  |  |  |
| The 28 day-mortality | 98 (14.06), n=697 | 77 (11.05), n=697 | 1.27 (0.95-1.72) | 0.11 |
| The length of ICU stays, d ^a^ | 2.72 (1.57-4.78), n=697 | 2.58 (1.44-4.87), n=697 | 0.07 (-0.22-0.36) | 0.63 |
| The length of hospital, d ^a^ | 7.96 (5.29-12.7), n=697 | 7.66 (4.98-13.4), n=697 | -0.11 (-0.80-0.58) | 0.75 |
| The duration of mechanical ventilation, d ^b^ | 0.78 (0.23-3.78), n=315 | 0.81(0.22-2.83), n=382 | 0.03 (-0.06-0.13) | 0.52 |
| The incidence of new mechanical  ventilation use during ICU stays, n (%) ^c^ | 114 (22.98), n=496 | 148 (30.27), n=489 | 0.69 (0.52-0.91) | **0.01** |
| The duration of vasopressors, d ^b^ | 0.88 (0.21-2.61), n=211 | 0.73(0.24-2.20), n=234 | 0.03 (-0.08-0.19) | 0.61 |
| The incidence of new vasopressors use during ICU stays, n (%) ^c^ | 49 (9.1%), n=486 | 45 (9.7%), n=463 | 0.92 (0.60-1.43) | 0.73 |

^a^ The significances between was calculated by paired Wilcoxon signed rank test because of the paired design.

^b^ Only including the patients with mechanical ventilation or vasopressors.

^c^ new vasopressors use or new mechanical ventilation use was defined as the first mechanical ventilation or vasopressors requirement after 24 hours of ICU admission.

^d^ Hazard ratio was reported for risk of 28-day mortality, odd ratios were reported for categorical variables, and differences between groups were reported for continuous variables.
